# Supplementary figures and images for: Irgm1-deficiency leads to myeloid dysfunction in colon lamina propria and susceptibility to the intestinal pathogen Citrobacter rodentium
Source: PLoS Pathog. 2020 May 26;16(5):e1008553. doi: 10.1371/journal.ppat.1008553 (PMC7274479; doi:10.1371/journal.ppat.1008553)

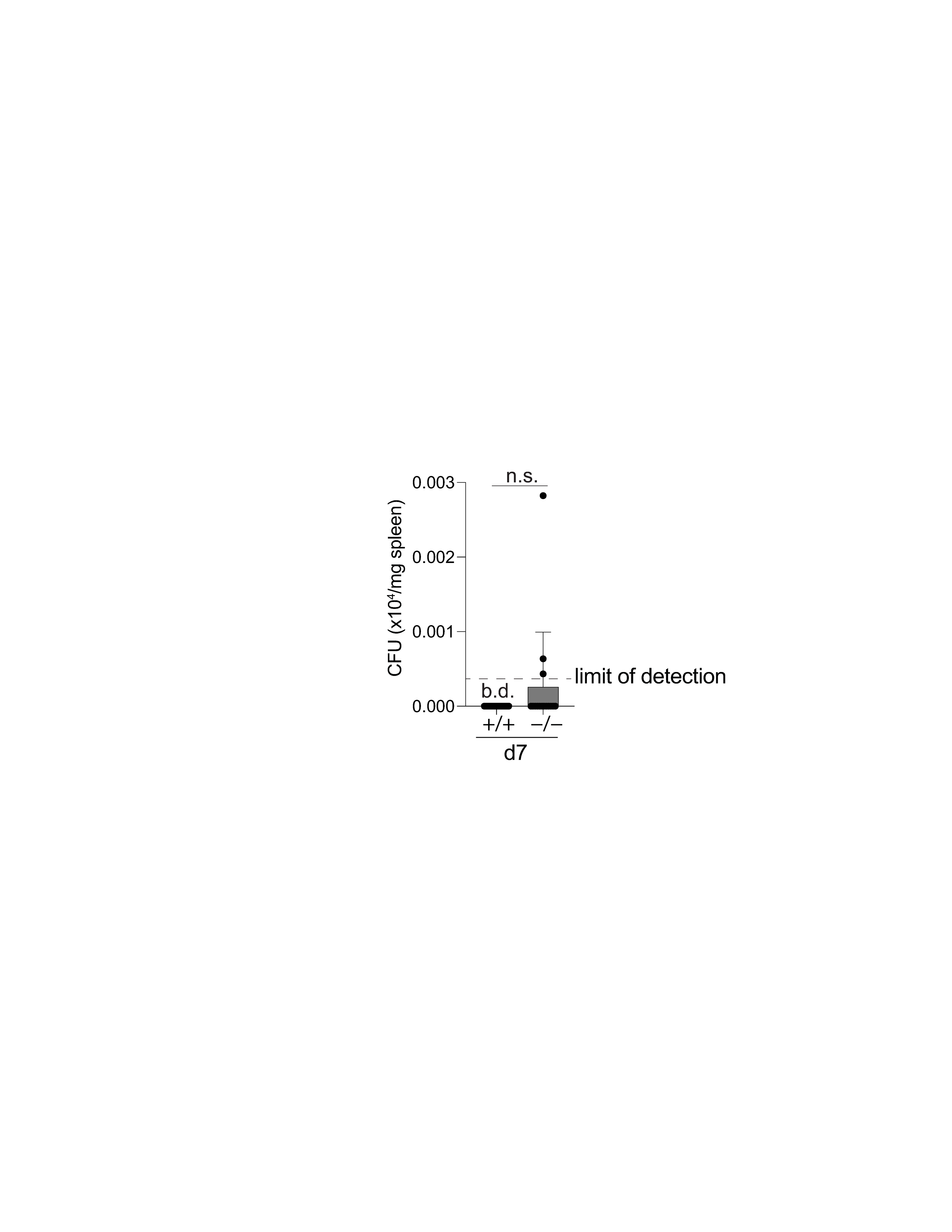

Supplement: S1 Fig — Data from n = 16 Irgm1+/+ mice and n = 15 Irgm1-/- mice were combined from two separate experiments. Error bars indicate mean ± SD. Unpaired Student’s t test; n.s., not significant; b.d, below detection. (TIF) [file ppat.1008553.s001.tif]

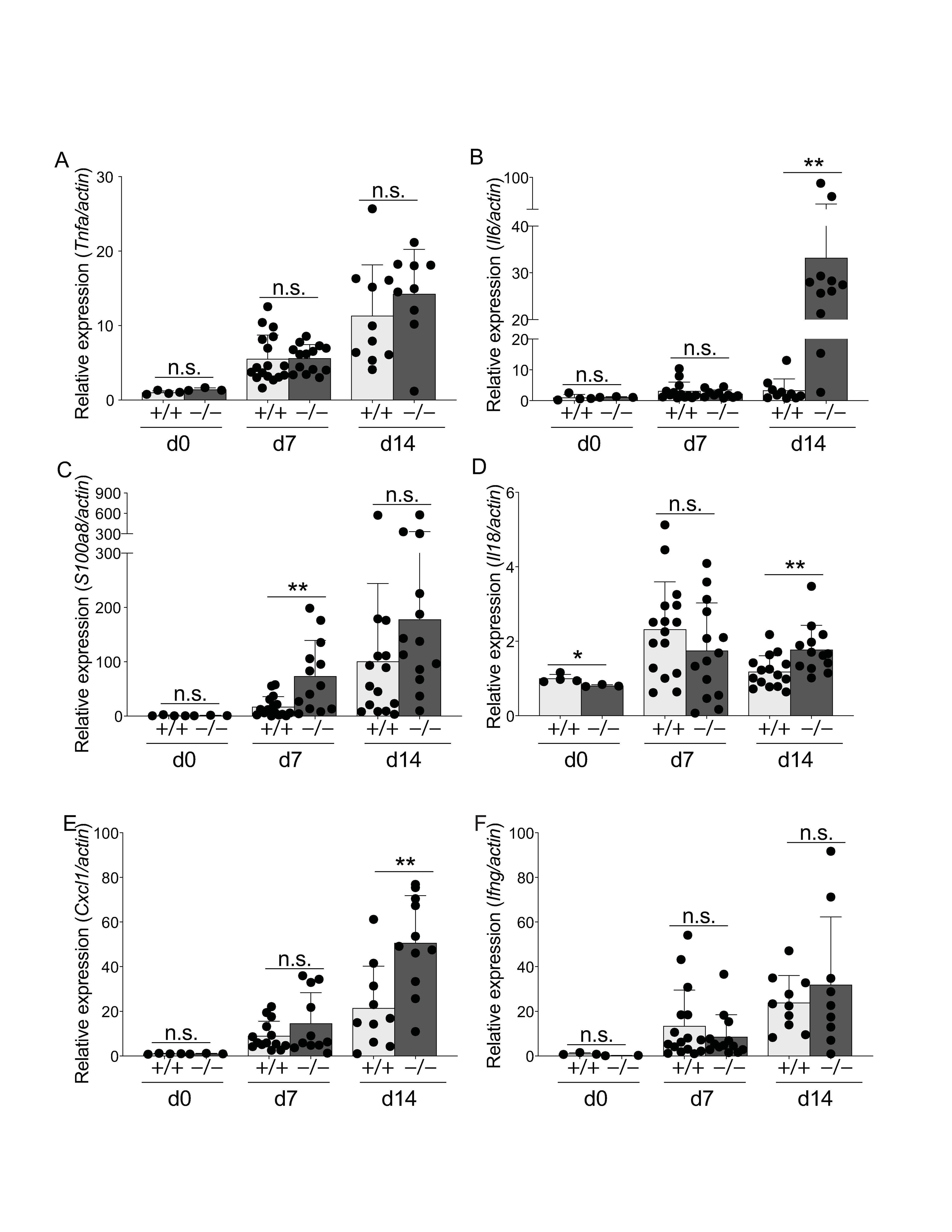

Supplement: S2 Fig — Separate cohorts of mice were analyzed at the indicated times for the abundance of mRNA expression of Tnfα (A), Il6 (B), S100a8 (C), Il18 (D), Cxcl1 (E), and Ifnγ (F) in distal colon. Data were combined from three independent experiments. Each dot represents one mouse; error bars indicate SD. *p < 0.05, **p < 0.01, ***p < 0.001, n.s., not significant (unpaired Student’s t test). (TIF) [file ppat.1008553.s002.tif]

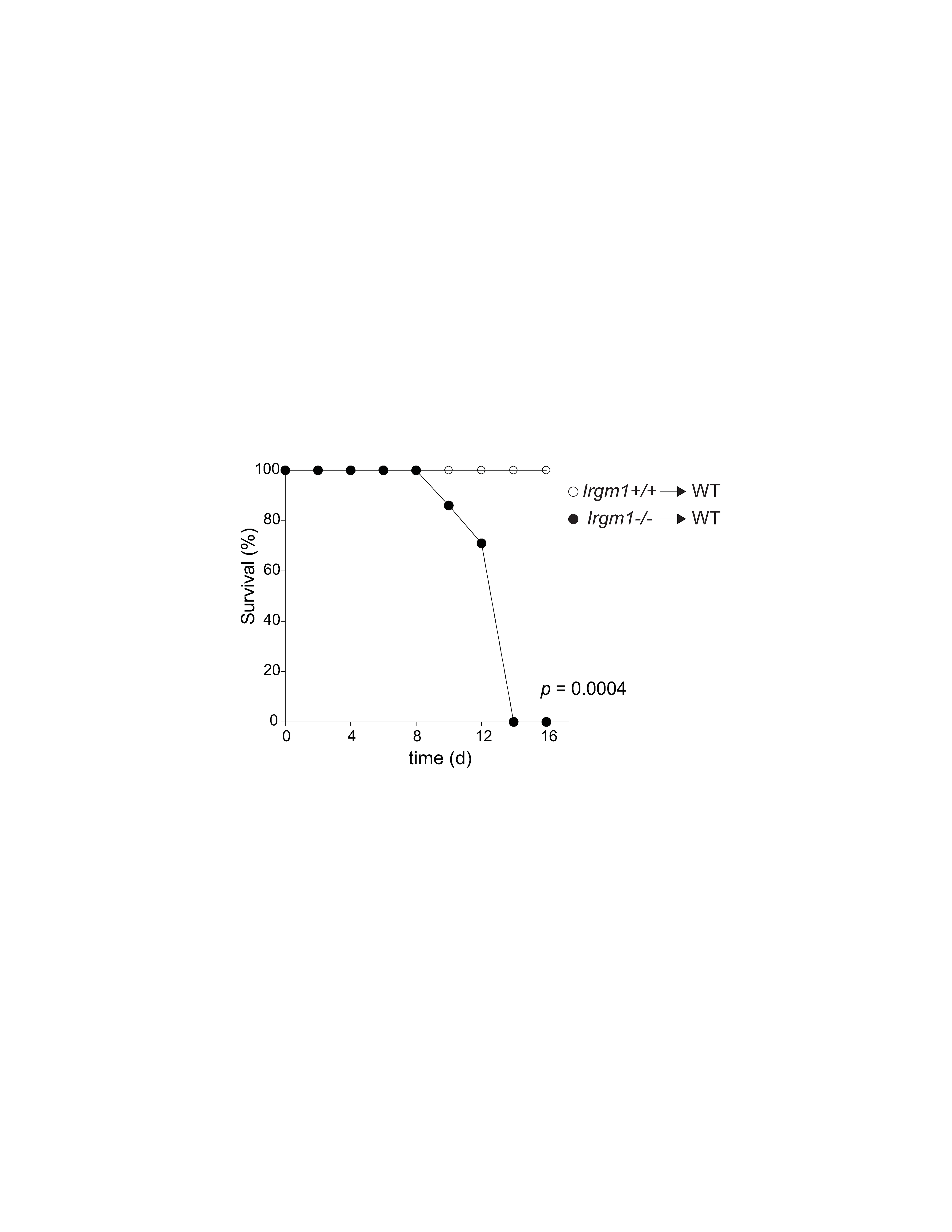

Supplement: S3 Fig — n = 7. The difference in survival was statistically significant [p = 0.0004, Log Rank (Mantel-Cox) test]. (TIF) [file ppat.1008553.s003.tif]

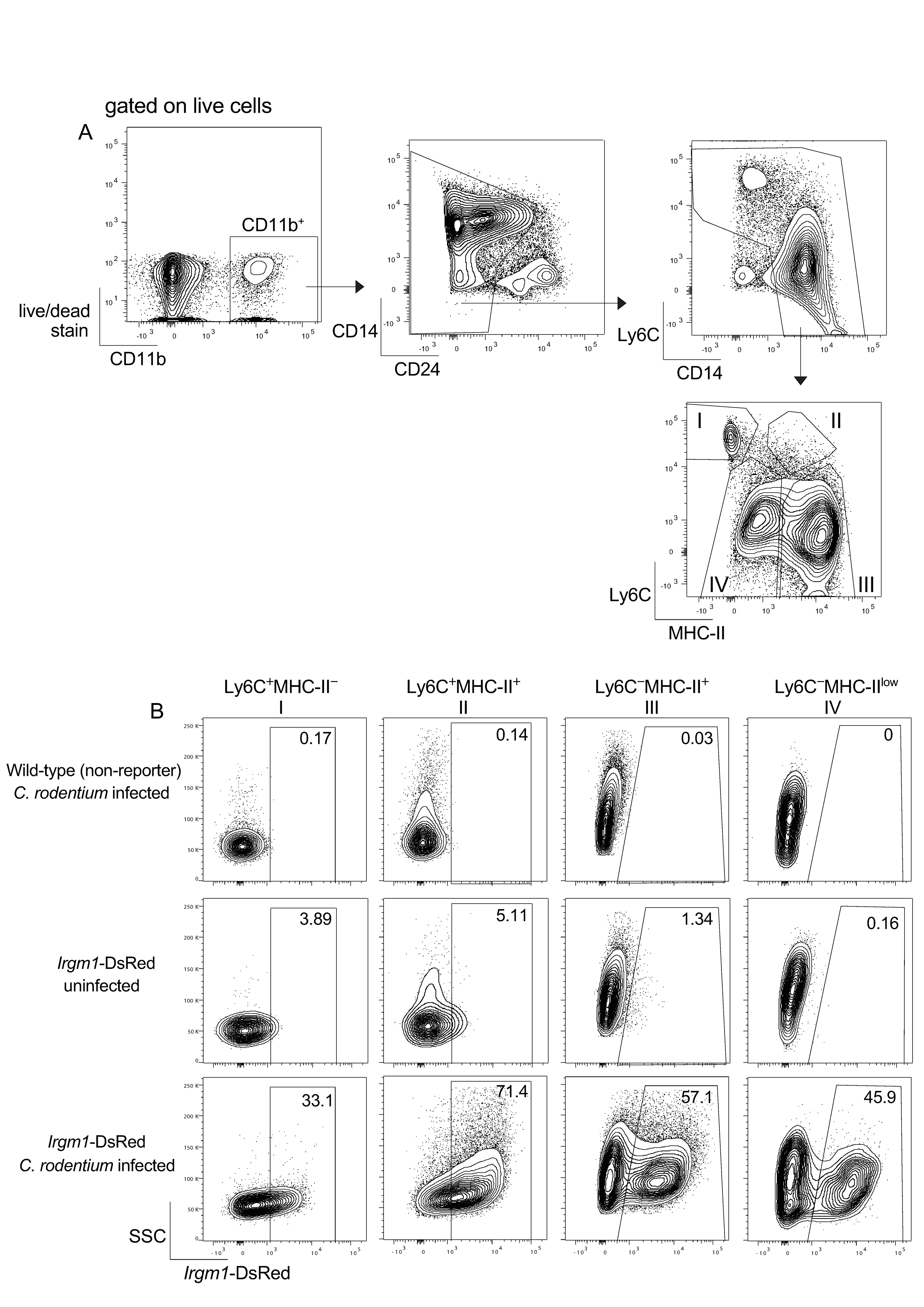

Supplement: S4 Fig — (A) After applying the indicated gates, populations were distinguished as follows: I. Monocyte (Ly6C+MHC-II-), II. transitioning monocyte (Ly6C+MHC-II+), III. macrophage (Ly6C-MHC-II+); IV. MHC-IIlo macrophage (Ly6C-MHC-IIlo). (B) C-LP monocyte/macrophage populations from mice of the indicated genotype were analyzed for expression of the Irgm1-DsRed reporter during steady-state or 10 days after C. rodentium infection. Data in (B) are representative of two independent experiments each including at least three mice of each genotype and condition. (TIF) [file ppat.1008553.s004.tif]

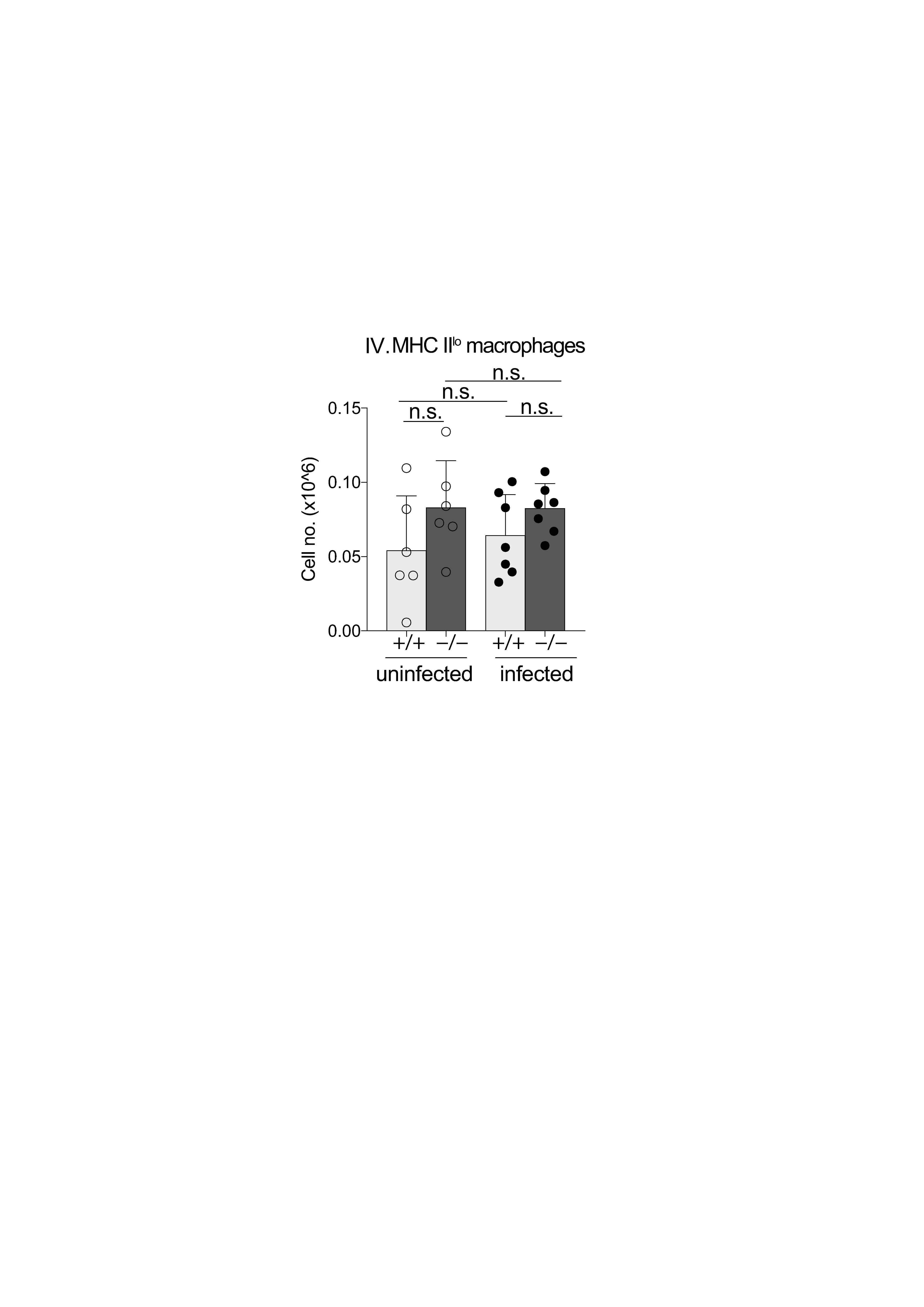

Supplement: S5 Fig — Data were combined from two independent experiments. Error bars represent mean ± SD. n.s., not significant (unpaired Student’s t test) (TIF) [file ppat.1008553.s005.tif]

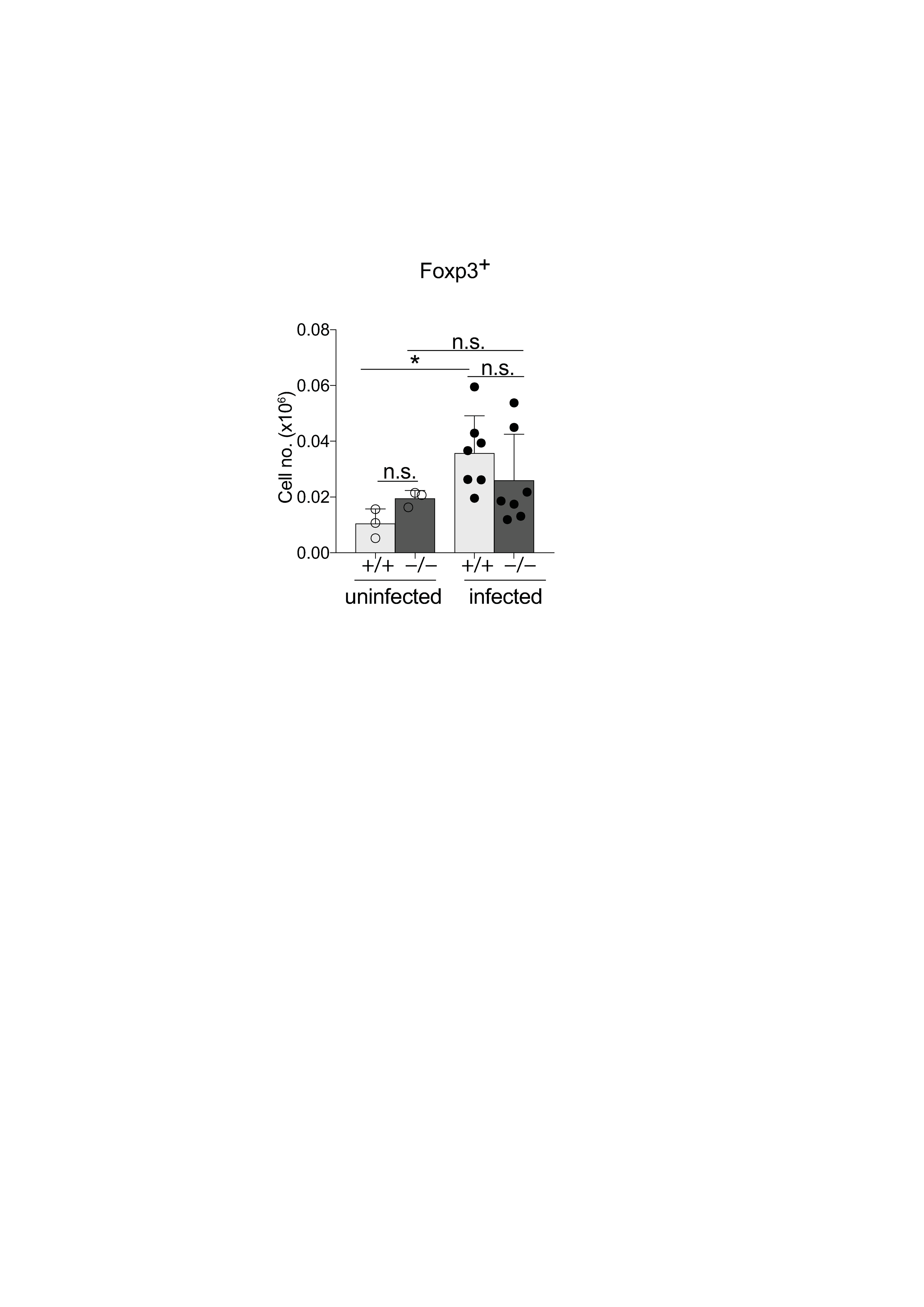

Supplement: S6 Fig — Data were combined from two independent experiments. Error bars represent mean ± SD. *p < 0.05, n.s., not significant (unpaired Student’s t test). (TIF) [file ppat.1008553.s006.tif]

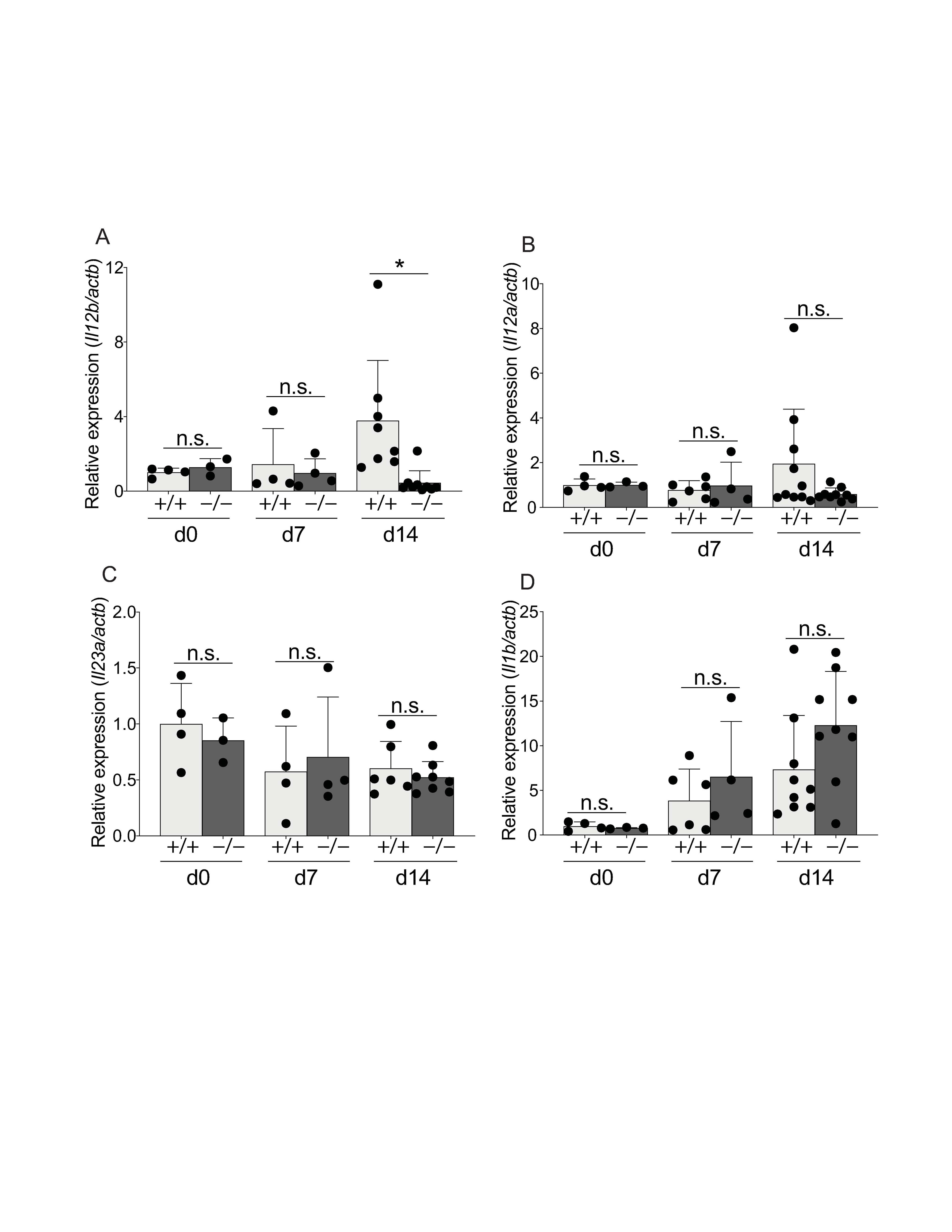

Supplement: S7 Fig — Distal colon from uninfected or C. rodentium infected mice described in Fig 2E–2G were analyzed for tissue expression of mRNA for IL-12b/p40 (A), IL-12a/p35 (B), IL-23/p19 (C), and IL-1β (D). Error bars represent mean ± SD. *p < 0.05, n.s., not significant (unpaired Student’s t test). (TIF) [file ppat.1008553.s007.tif]

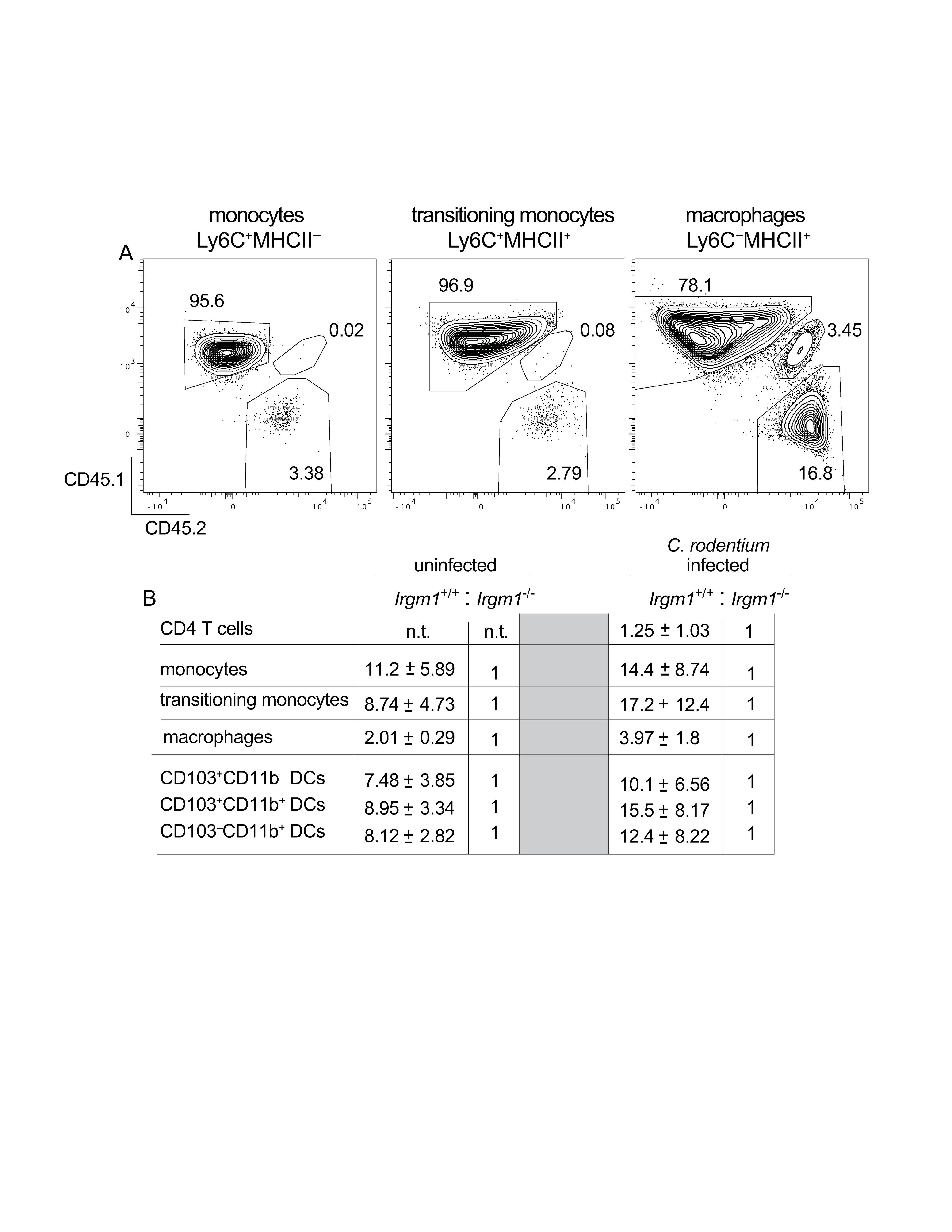

Supplement: S8 Fig — (a-b) Mixed hematopoietic chimera described in Fig 6 were infected with C. rodentium for 10 days and analyzed for C-LP immune cells originating from Irgm1+/+ (CD45.1), Irgm1-/- (CD45.2), or host-derived (CD45.1+CD45.2+) cells. (A) Representative flow plots showing the relative abundance of host or donor derived cells among C-LP monocytes, transitioning monocytes, and macrophages in mixed hematopoietic chimera infected with C. rodentium. (B) The ratio of Irgm1+/+ to Irgm1-/- cells among the indicated cell type in C-LP of mixed hematopoietic chimera, either uninfected or infected for 10 days with C. rodentium. Data represents mean ± SD. n.t., not tested. (TIF) [file ppat.1008553.s008.tif]

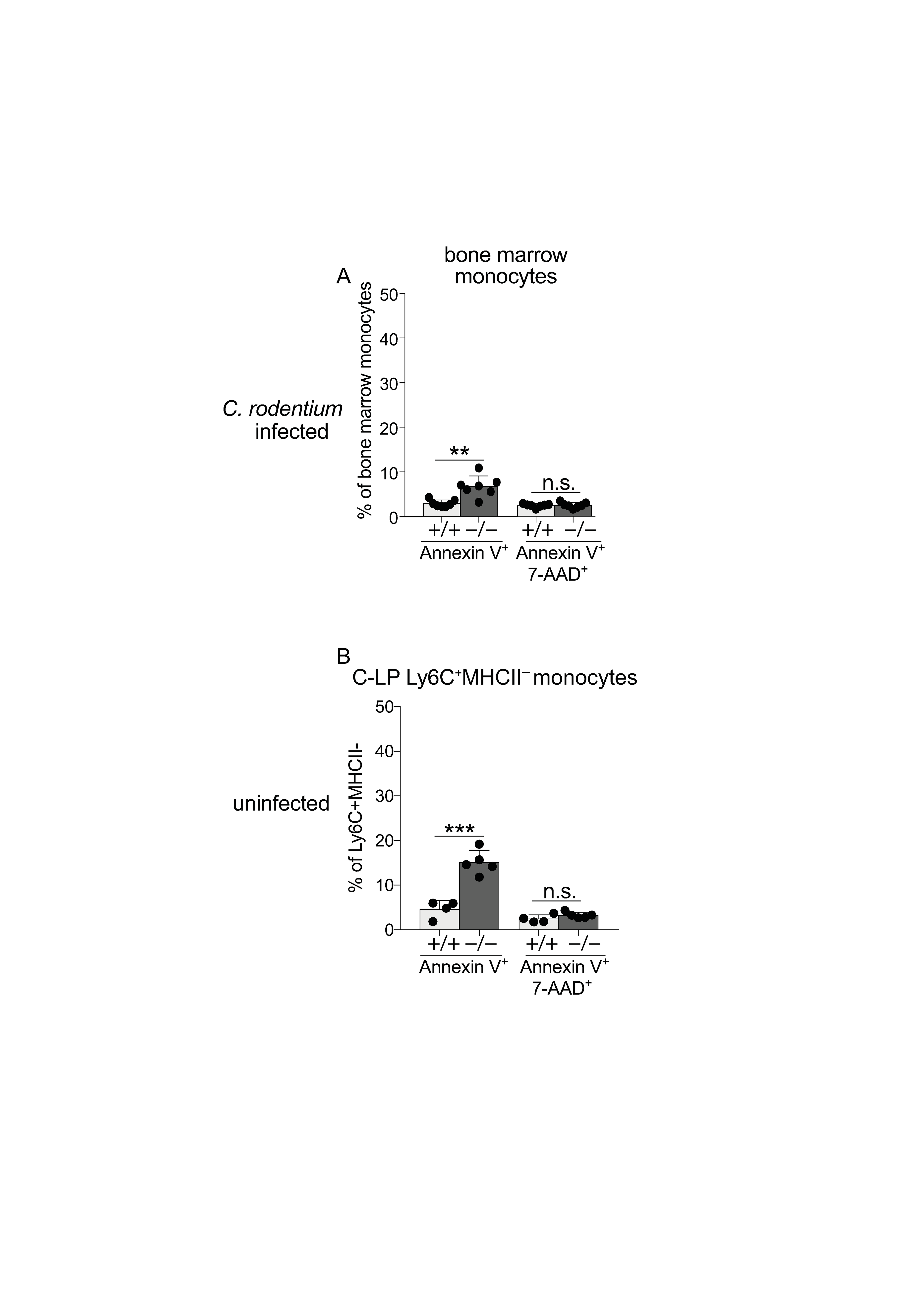

Supplement: S9 Fig — (A) C. rodentium infected mice described in Fig 7 were analyzed for the percentage of Annexin V+ or Annexin V+7AAD+ cells among bone marrow monocytes. (B) C-LP monocytes from uninfected mice of the indicated genotype were analyzed as above. Each dot represents one mouse. Error bars represent mean ± SD. **p < 0.01, ***p < 0.001, n.s., not significant (unpaired Student’s t test). (TIF) [file ppat.1008553.s009.tif]

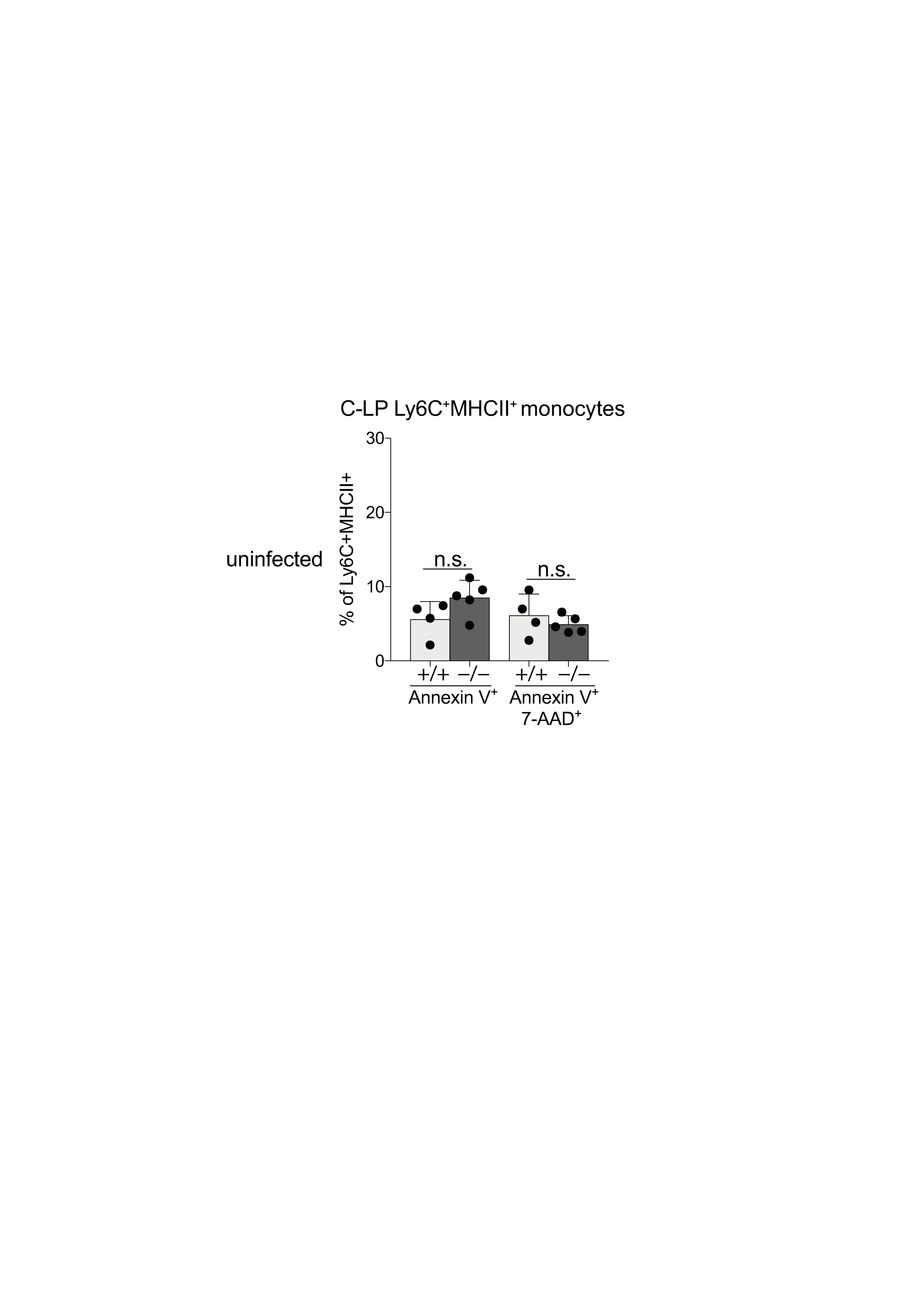

Supplement: S10 Fig — Each dot represents one mouse. Error bars represent mean ± SD. n.s., not significant (unpaired Student’s t test). (TIF) [file ppat.1008553.s010.tif]

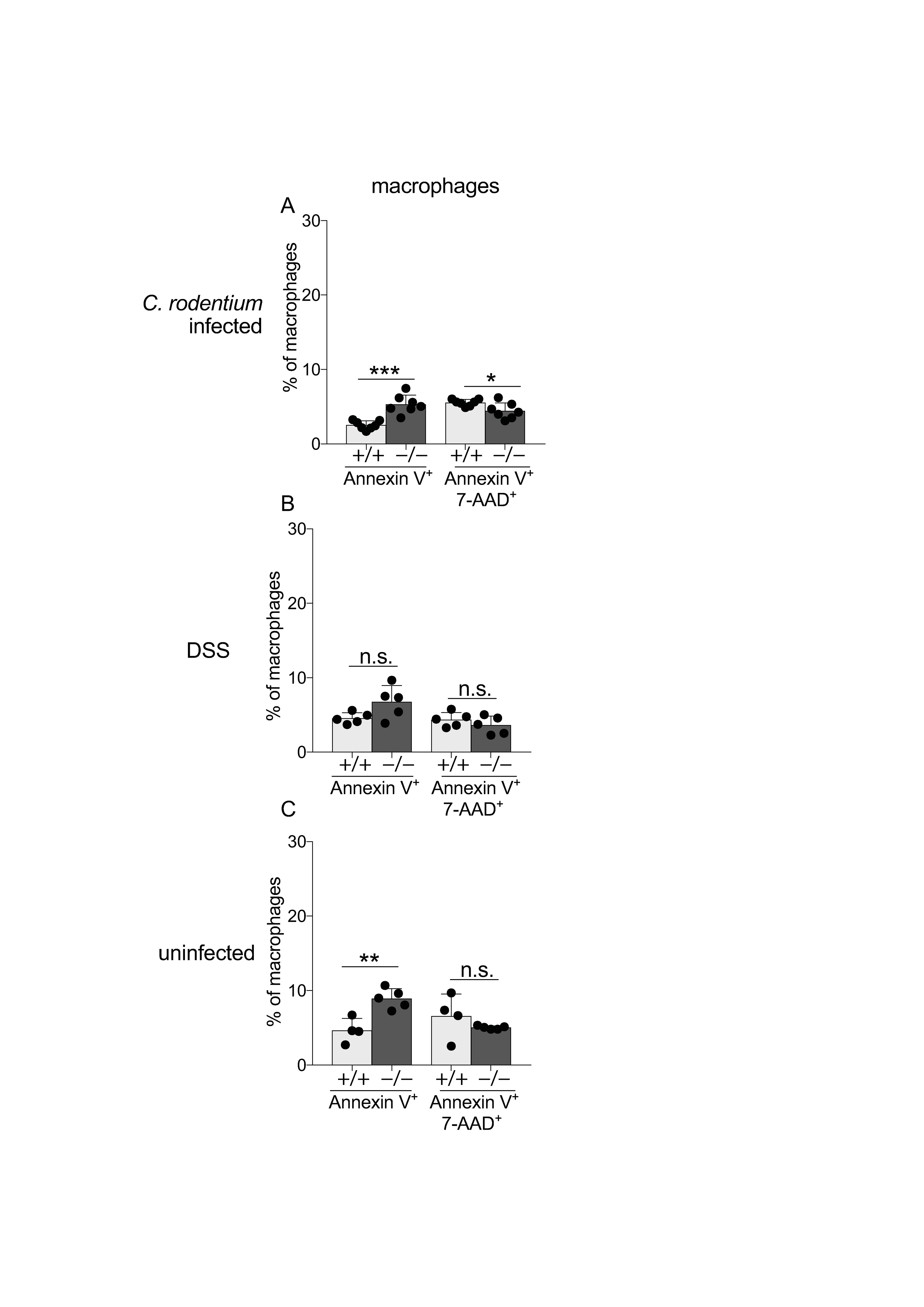

Supplement: S11 Fig — The mice of the indicated genotype were C. rodentium infected (A) DSS treated (B) or uninfected mice (C). Each dot represents one mouse. Error bars represent mean ± SD. *p < 0.05, **p < 0.01, ***p < 0.001, n.s., not significant (unpaired Student’s t test). (TIF) [file ppat.1008553.s011.tif]

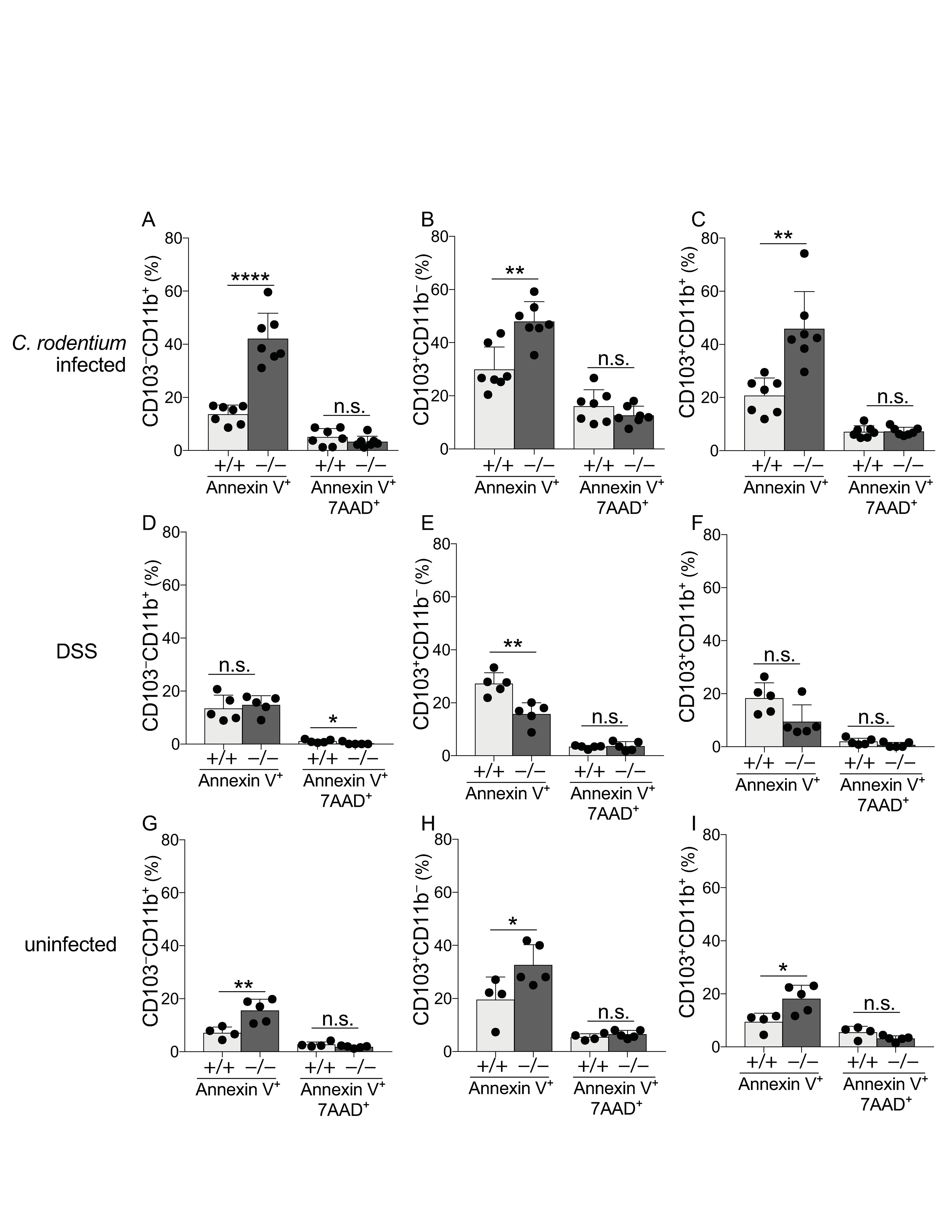

Supplement: S12 Fig — The percentage of Annexin V+ or Annexin V+7AAD+ cells among C-LP CD103-CD11b+ DCs (A), CD103+CD11b- DCs (B), and CD103+CD11b+ DCs (C) in C. rodentium infected mice described in Fig 7. C-LP DCs from DSS treated mice (D-F) or uninfected/untreated mice (G-I) were analyzed as above. Error bars represent mean ± SD. *p < 0.05, **p < 0.01, ****p < 0.0001, n.s., not significant (unpaired Student’s t test). SD. *p < 0.05, **p < 0.01, ****p < 0.0001 (unpaired Student’s t test). (TIF) [file ppat.1008553.s012.tif]
